# Key Parameters for Performance and Resilience Modeling of 3D Time-of-Flight Cameras Under Consideration of Signal-to-Noise Ratio and Phase Noise Wiggling

**DOI:** 10.3390/s25010109

**Published:** 2024-12-27

**Authors:** Niklas Alexander Köhler, Marcel Geis, Claudius Nöh, Alexandra Mielke, Volker Groß, Robert Lange, Keywan Sohrabi, Jochen Frey

**Affiliations:** 1 Department of Health, University of Applied Sciences Mittelhessen, 35390 Giessen, Germany; marcel.geis@ges.thm.de (M.G.); claudiusnoeh@googlemail.com (C.N.); volker.gross@ges.thm.de (V.G.); 2 Institute of Safety and Security Research (ISF), Hochschule Bonn-Rhein-Sieg, 53757 Sankt Augustin, Germany; alexandra.mielke@smail.emt.h-brs.de (A.M.); robert.lange@h-brs.de (R.L.); 3 Department of Electrical Engineering and Information Technology, University of Applied Sciences Mittelhessen, 35390 Giessen, Germany; jochen.frey@ei.thm.de

**Keywords:** Time-of-Flight (ToF), amplitude wiggling, phase noise wiggling, absolute phase wiggling, camera modeling, camera characterization, sensor resilience

## Abstract

Because of their resilience, Time-of-Flight (ToF) cameras are now essential components in scientific and industrial settings. This paper outlines the essential factors for modeling 3D ToF cameras, with specific emphasis on analyzing the phenomenon known as “wiggling”. Through our investigation, we demonstrate that wiggling not only causes systematic errors in distance measurements, but also introduces periodic fluctuations in statistical measurement uncertainty, which compounds the dependence on the signal-to-noise ratio (SNR). Armed with this knowledge, we developed a new 3D camera model, which we then made computationally tractable. To illustrate and evaluate the model, we compared measurement data with simulated data of the same scene. This allowed us to individually demonstrate various effects on the signal-to-noise ratio, reflectivity, and distance.

## 1. Introduction

The native systematics of ToF camera systems according to the amplitude-modulated continuous wave (AMCW or indirect ToF) method can be simply described by two typical properties: first, a statistical measurement uncertainty that significantly depends on the intensity of the received signal [1,2,3,4]; and second, (phase-) wiggling, a systematic reproducible measurement error that depends on the phase of the received return signal [5,6,7,8,9].

In this paper, we will demonstrate through simulations and measurements on a large variety of distances and signal intensities that the previous assumptions are incomplete and both properties must be considered coherently. More precisely, the amount of range noise is also subject to wiggling (phase noise wiggling in contrast to absolute phase wiggling). While systematic measurement errors can be compensated for by calibration, the wiggling of the range noise remains an inherent property in the measurement system. Therefore, it should be incorporated into the ToF model.

To date, it has been recognized in numerous publications how the statistical measurement uncertainty of ToF cameras depends on the SNR of the system. The term “signal” refers to the total number of collected photons gathered in each pixel through active illumination, assuming 100 percent modulation depth in illumination and pixel demodulation. Especially at high frequencies (i.e., those exceeding 50 MHz), this often corresponds to only a reduced part of the active illumination due to bandwidth limitations. In many cases, the noise is dominated by the shot noise of the electrons generated in the pixel [10]. When used outdoors, the background light is often dominant here, whereas indoors, the shot noise of the active illumination itself is limiting. The correlations are described in more detail in [1,3].

This property of ToF cameras offers a great advantage over other measurement methods, such as stereo vision or structured light. Previous work demonstrates that ToF camera systems show resilience to various effects such as interfering light sources or changing reflection factors [11]. However, significant differences in performance can occur between individual systems [11,12,13]. Uncertainty of the charge carriers generated in the pixel is determined; then, for each ToF measurement, in addition to the distance value itself, its uncertainty can also be stated directly, without the need for statistical analyses. Previous work [1,2,3,8,14,15,16] assumes, in this context, that this distance noise only depends on the described signal intensity and noise value, but we will show that the distance value itself, or the phase position of the AMCW measurement, has an additional systematic influence on the measurement uncertainty. Zheng et al. already recognized that wiggling also influences the statistical error in addition to the systematic error [9]. However, the additional influence of the distance dependence of the signal intensity is neglected here, and the underlying model assumes ToF pixels as analog mixers in a simplified way. In our simulation, we consider both photon shot noise and sensor noise, as well as the digital short-time-integrating properties of ToF pixels, which differ from an analog mixing property.

## 2. ToF Camera Model

**Figure 1:**
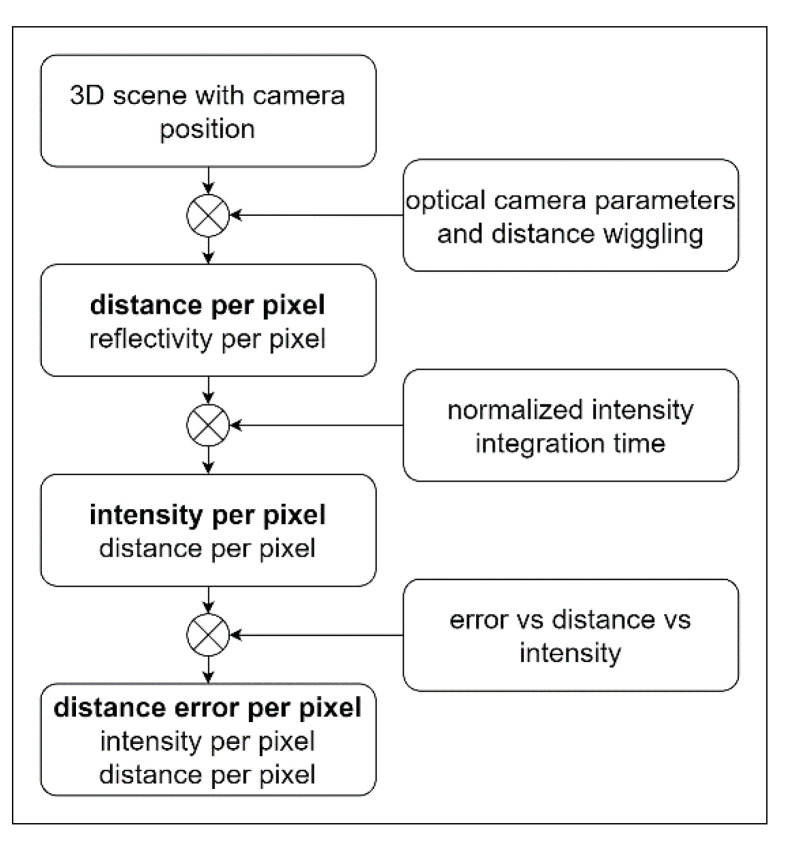
Proposed 3D camera model. The data from the modeled scene (box at the top) are processed step by step with the experimentally determined camera parameters (boxes on the right side). Finally, a complete dataset per pixel (distance error, intensity, and distance) is obtained.

The camera parameters presented in this work can be used for a generic model of a 3D camera. In our experiments, we used the flexx2 3D camera from pmdtechnologies AG, Siegen, Germany. Even if it can be assumed that the parameters determined for the model are camera-specific, the methodology for camera modeling presented here should also be transferable to other ToF cameras, in particular, the independent recording of intensity, distance, and the implementation in a lookup table. In Figure 1, our proposed model is depicted. Starting from the modeled 3D-scene, the relevant parameters, namely distance, intensity, and distance error, are determined sequentially for each pixel.

In the first step, starting from the scene and the camera’s position in space, the distance and reflectivity of the object area seen by each pixel are determined. This can be accomplished using a pinhole approach or a more accurate description of the lens properties.

In the second step, based on this pixel information, as well as the normalized intensity distribution over the sensor array and the exposure time, the intensity of the pixel signal is determined. In this process, pixels that are in saturation may also be marked as invalid.

In the final step, the error for each pixel is calculated using the intensity and distance. By considering distance, this can account for not only the quadratic decay over intensity but also the error wiggling as described in this paper. The final dataset per pixel consists of distance, intensity, and distance error in the form of a standard deviation.

These output data can be used for evaluating an application or, in a subsequent step, for generating simulated 3D video sequences with noisy distance information.

A key challenge in experimentally demonstrating the effect of phase noise wiggling is the simultaneous attenuation of signal amplitude with increasing distance, making it difficult to separate the influence of phase from amplitude. To address this, we conduct over one billion pixel measurements, varying exposure time and measurement distance. This extensive dataset allows clear identification and quantification of the phase noise wiggling effect. Measurements were performed without ambient light on a white diffuse target with Lambertian characteristics to ensure reproducible conditions. The setup allows independent variation in the received amplitude, controlled through exposure time, and the phase position of the signal, adjusted by target distance.

In typical illuminated interiors (e.g., LED lighting or fluorescent tubes), the spectral characteristics of these light sources, combined with the narrowband optical filters of ToF cameras, ensure that the shot noise from the background illumination does not dominate the noise of the distance measurements. The results of the measurements can therefore also be transferred to indoor applications.

Another point concerns the internal filtering algorithms in the cameras. These were disabled for all measurements, and the model does not take them into account. Therefore, it is up to the user to filter the output signals separately after the simulations.

## 3. Experimental Setup

**Figure 2:**
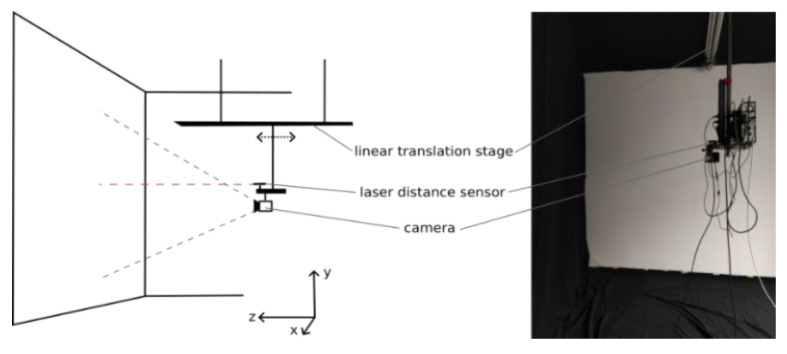
Experimental set-up. (**Left**): Schematic layout of the measuring environment. (**Right**): Photo of the actual measurement.

**Figure 3:**
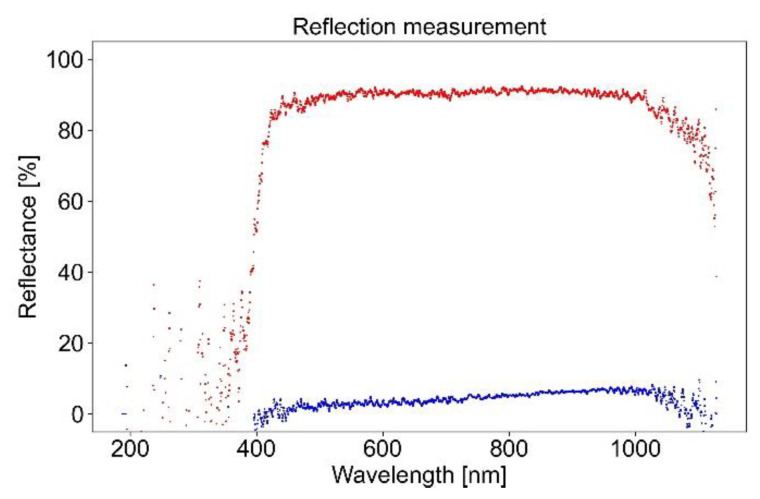
Reflectivity of the target wall (red dots) and the curtains (blue dots) measured at an angle of 90°.

The measurements took place in a controlled measurement environment. To ensure a standardized measurement environment and to minimize the effects of interfering light, the measurements were carried out in a dark environment. As shown in Figure 2, the 3D camera flexx2 is mounted on a linear motion rail. The flexx2 is aligned along the *z*-axis. The image scene consists of a white wall, which also serves as the depth-measurement target. The average reflectivity of the measurement target is approximately 90% (cf. Figure 3).

To adjust the distance between target and camera, the camera is attached below a linear motion rail, which moves along the *z*-axis (Figure 2).

Although the distance can be measured indirectly by moving the camera along the motion rail, a laser distance sensor measures the absolute distance between the camera stage and the wall. The laser is of type DAE-10-050 from Dimetix AG, Herisau, Switzerland with a precision of 1 mm (2 σ) [17] and is also attached to the rail. Furthermore, to reduce scattered light and reflections, all other walls are covered with a molton fabric of 10% reflectivity compared to the target wall (Figure 3). A SILVER Nova Super Range TEC Spectrometer from StellarNet Inc., Tampa, FL, USA was used to perform the reflection measurements [18].

For a single measurement, the camera is moved from 0.37 m to 3 m along the linear translation stage. The distance was defined based on the limitation of the translation stage and the unambiguous range of the camera. Every 10 cm, the translation stage stops, and 100 frames are taken by the camera. A total of 28 measurement positions were thus performed. The frames taken at a single position are then averaged into a single file. This includes averaging the depth data and the intensity data. To ensure that invalid pixels do not corrupt the data, they are not taken into account for averaging data or calculating the standard deviation, and the number of invalid values is saved pixel-wise.

To generate a high quantity of different distance–intensity values, measurements were taken using ten different exposure times offered by the pmd flexx2 (cf. Table 1).

## 4. Experimental Results

### 4.1. Independent Pixel Behavior

**Figure 4:**
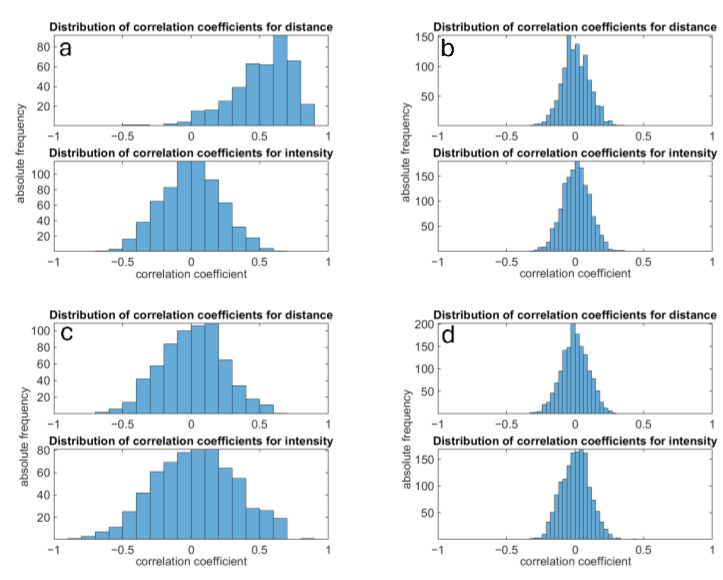
Histograms of correlation coefficients between adjacent pixels. Histogram (**a**): filters enabled and low light condition; histogram (**b**): filters disabled and low light condition; histogram (**c**): filters enabled and high light condition; Histogram (**d**): filters disabled and high light condition. For characterization and modeling, it is crucial that the pixel signals are independent of each other, meaning no correlation is present. Without filters, the flexx2 fulfills this condition. All following measurements in this paper are made without filtering.

For successful modeling, it is vital that the signals and errors of individual pixels remain independent. Adjacent pixels should not influence one another; instead, the signals should depend solely on the camera’s electro-optical characteristics and the observed scene. Initially, any correlations between individual pixels are examined. Ideally, the correlation coefficients should average around zero, indicating independent behavior. The results of this analysis are depicted in Figure 4. The use of digital image processing filters, such as smoothing and hole-filling filters, leads to partial correlation among neighboring pixels. This occurs because, especially at low intensities, averaging over neighboring pixels results in shared information components. To avoid such correlation, the filters were deactivated for these measurement series. The observed correlation distribution for measurements without filters with a mean value of zero (cf. Figure 4b,d) is a strong indicator that there is no correlation between the pixels.

### 4.2. Normalized Intensity

**Figure 5:**
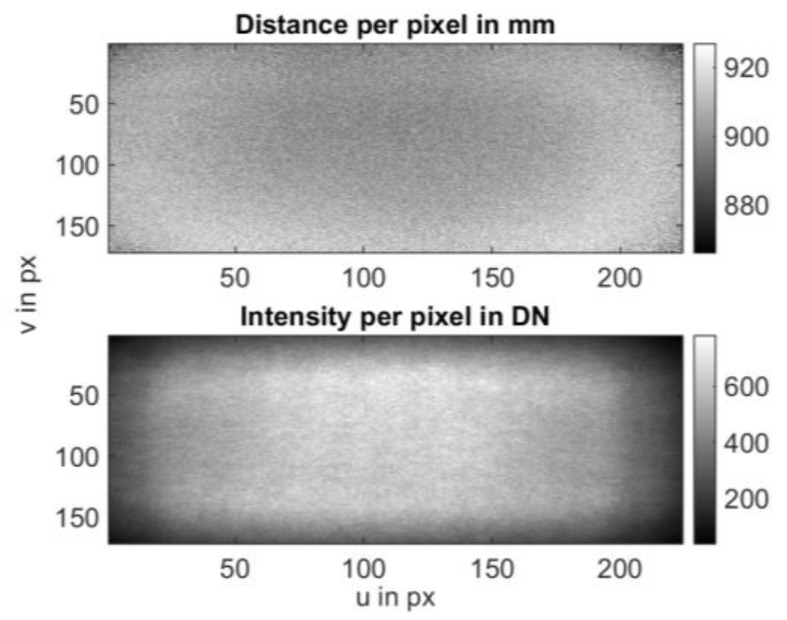
Analysis of 100 measurements on a flat target with constant reflectivity. **Top**: Mean distance value. Apart from the fixed-pattern noise, no further anomalies are evident. **Bottom**: Mean amplitude value. The distribution arises from the illumination characteristics and the detector optics in conjunction with the sensor array. This characteristic pattern can be normalized and used for the camera model.

When observing a scene through a 3D camera, the recorded pixel intensity is influenced by several factors, including distance, exposure time, and reflectivity. However, even if all pixels encounter identical object conditions (such as consistent distance and reflectivity, like a flat surface with uniform reflectivity) and share the same exposure duration, they will register varying intensities based on their location within the sensor array. This discrepancy arises from multiple elements, including the increasing radial distance from the sensor’s periphery, lens characteristics, and notably, the illumination conditions (refer to Figure 5 for clarification).

To account for this, a relative intensity distribution over the sensor array can be determined. This distribution indicates how pixel intensities behave relative to each other under otherwise constant conditions.

**Figure 6:**
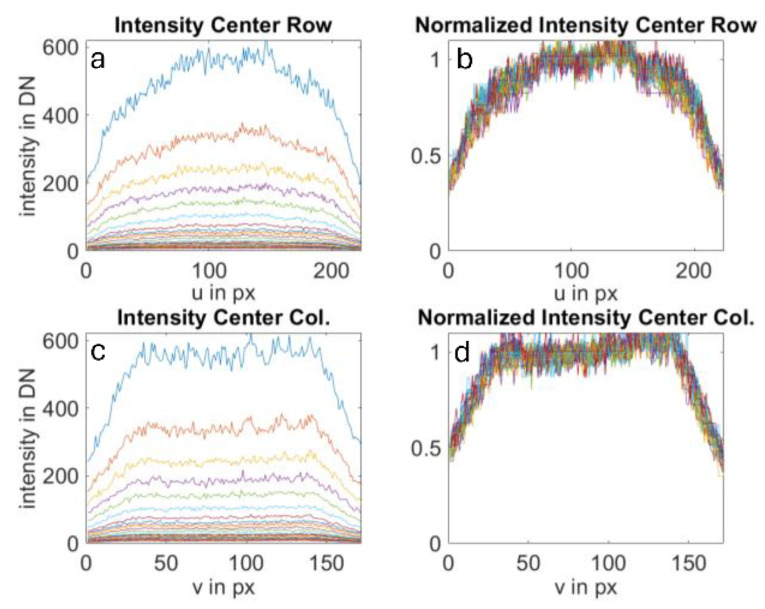
Intensity profiles (diagrams (**a**,**c**)) at various distances. Normalized intensities (diagram (**b**,**d**)) exhibit significant overlap. As a result, a normalized intensity can be used for all distances.

To utilize this relative intensity distribution in the camera model, it must be ensured through investigations that this distribution is at least independent of the distance to the object plane. To achieve this, we conducted 100 measurements at different distances on a flat object, determining the mean intensity for each pixel. In Figure 6, the absolute intensity profiles for a middle row and column of the sensor array are visible on the left side. It is evident that intensities decrease with increasing distance, while the relative intensity profile remains nearly constant. The profile only deviates at short distances. This can be explained by the fact that central pixels slowly saturate at these distances, distorting the intensity profile. On the right side, the profiles were normalized to the brightest area, making them more comparable. The curves closely align (except for the curve with saturation effects). Therefore, it can be inferred from this series of measurements that a normalized intensity distribution is a suitable means for modeling camera behavior.

**Figure 7:**
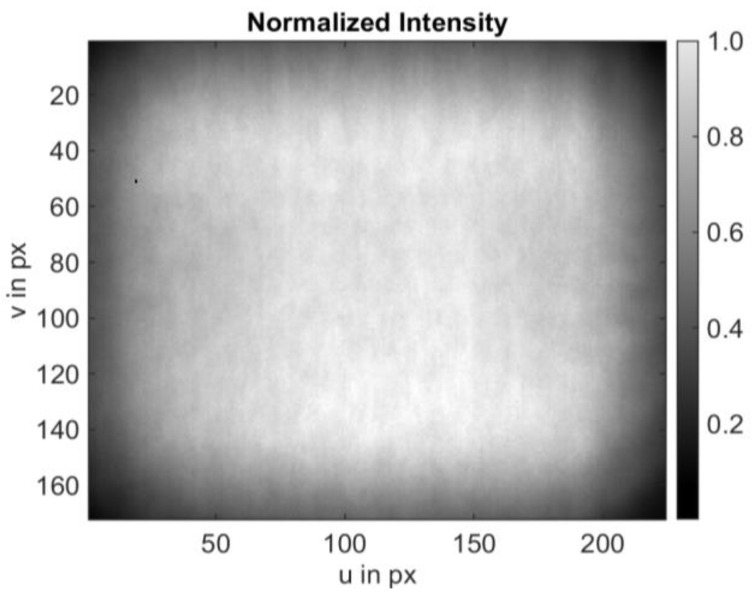
Normalized intensity of the sensor array. An intensity value of 1 is attributed to the brightest region on the sensor. This region is not necessarily required to be at the center of the array.

In Figure 7, the normalized intensity profile of the entire sensor is depicted. The normalization was performed around the brightest pixel, which is assigned the value 1.

### 4.3. Intensity Versus Integration Time and Distance

**Figure 8:**
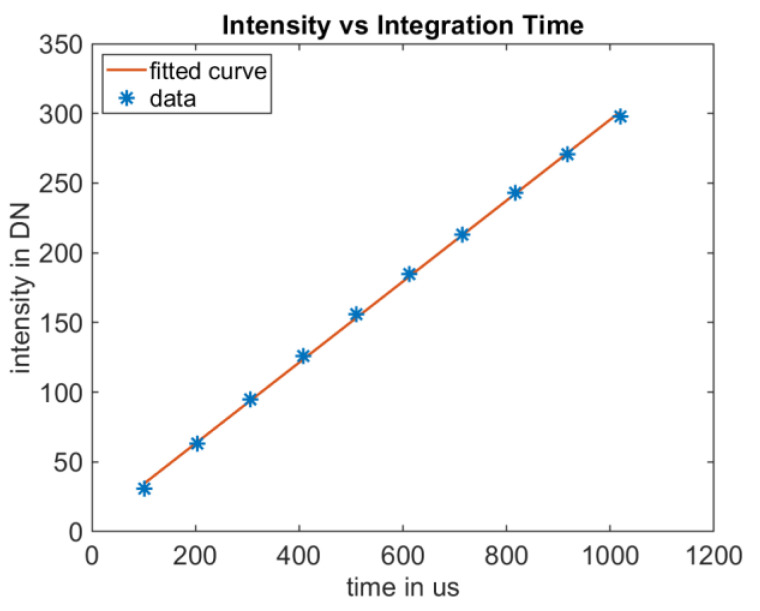
Intensity versus integration time of the center pixel. Intensity varies linearly with exposure time. This is valid until saturation is reached.

Due to the identical structure of the pixels in the sensor array, when examining the intensity profile versus exposure time, it is sufficient to analyze a single pixel. In this paper, we utilize the center pixel. Since pmdtechnologies sensors are linear image sensors without compression properties [19], the expectation is a linear relationship. This also applies when using suppression of background intensity (SBI), as in this case the pixel’s difference signal, which contains the distance information, is not compressed [3]. As shown in Figure 8, the measurements confirm this assumption. This applies until a pixel reaches saturation.

**Figure 9:**
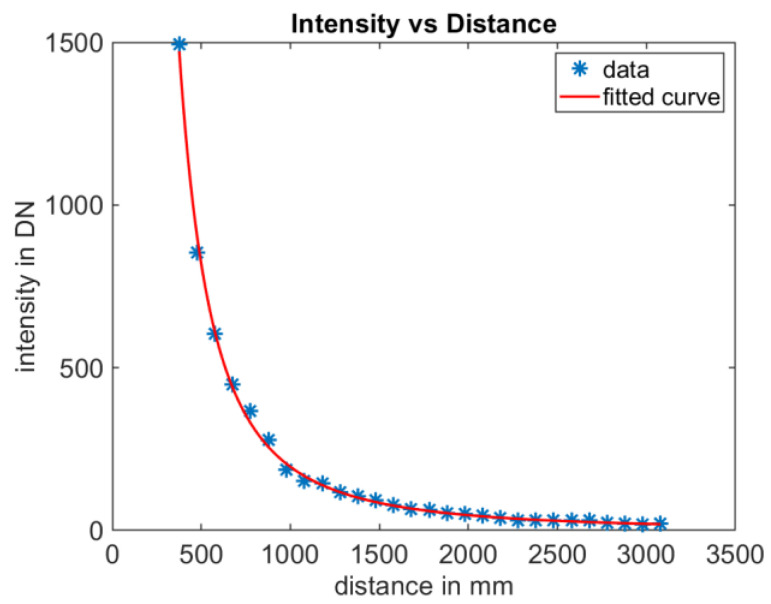
Intensity versus distance of the center pixel. Intensity varies as expected with distance. This is valid until saturation is reached.

The flexx2 camera is a system with active illumination. Due to its compact design relative to typical distances to the target, the illumination and the sensor can be considered to be placed at the same spatial point. Therefore, the relative intensity profile between pixels at different distances from the same target (monochromatic flat surface) remains unchanged. For example, areas with low illumination are always observed by the same groups of pixels (cf. edge pixels in Figure 7). However, the absolute intensity values of the pixels decrease with the square of the distance d. As in the previous analysis, the center pixel is used here again. In Figure 9, the measurement results and a fitted curve of the intensity i are presented. The curve is parameterized with these values:

$$i=3.047\cdot 10^{8}\cdot {\left(\frac{d}{\mathrm{mm}}\right)}^{-2.064}$$

The determined curve closely approximates the theoretically expected behavior.

### 4.4. Statistical Error Versus Distance and Intensity

**Figure 10:**
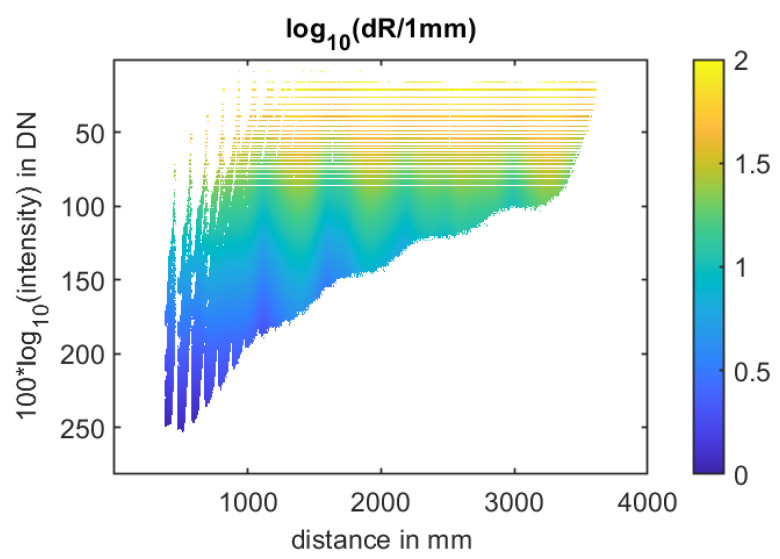
Statistical error (distance noise) versus distance and intensity. The error decreases with increasing intensity and shows a strong dependency on distance (phase noise wiggling). As the signal intensity decreases with distance, the dataset is incomplete for low intensities at short distances. The same applies to long distances. Due to technical limitations, the shortest distance measured is 0.37 m.

To investigate the dependence of the error on intensity and distance, 28,000 frames, totaling 1064 Mio. pixel values, were evaluated. These measurement series vary in distance and integration time (cf. Section 3). For each set of measurements, comprising a total of 100 measurements, intensity, distance, and distance error in the form of standard deviation are determined for each pixel. Each dataset is then processed in a multidimensional array. The x and y coordinates correspond to intensity and distance, and in the other two dimensions, the error is accumulated, or the number of pixels processed in this coordinate is increased by 1. After processing all the measurement series and subsequently calculating the average error per coordinate, the depicted results are obtained (cf. Figure 10).

The error decreases with increasing intensity following a 1/N dependency and, with higher intensities, a 1/sqrt(N) dependency. This behavior has been extensively discussed in [5,7]. The transition from 1/N to 1/sqrt(N) is subject to the ratio of intensity-dependent photon shot noise to constant electrical noise. However, these physical phenomena do not consider the distance or the phase relationship of the electrical modulation signal to the optical signal at the sensor.

Nonetheless, our investigations reveal a systematic dependence of the distance noise on both the distance-dependent SNR and the phase of the received optical signal. The previous literature (i.e., [5,8]) indicates that employing a four-phase algorithm results in a systematic error in determined distance, known as “wiggling”. Through simulations and extensive measurements across a broad range of distances and signal amplitudes, we have shown that wiggling not only contributes to systematic distance errors but also introduces periodic variations in statistical measurement uncertainty that are further superimposed on the SNR dependence. Our dataset provides a valuable lookup table for modeling (cf. Section 6). The subsequent section details the statistical error attributable to wiggling.

## 5. Wiggling

Using a simple MATLAB simulation (The MathWorks Inc., Natick, MA, USA, (R2023a) Version 9.14) we first determine the expected pixel values in terms of number of electrons for phase offsets of the demodulator of 0, 90, 180, and 270 degrees for a four-phase process of an idealized two-tap ToF pixel with an assumed full-well capacity of 100,000 electrons. These pixel values are then corrupted artificially by the shot noise of the measurement, and finally the measured phase is calculated from the artificially noisy pixel values using the ATAN function. In a higher-level loop, the received signal is then varied from 0 degrees to 360 degrees. An additional superordinate loop repeats this simulation (for example, 5000 times). This creates a database with which the expected mean value and the expected reproducibility of the measured value can be simulated for each phase position. The demodulation is assumed to be perfect, without bandwidth limitations.

**Figure 11:**
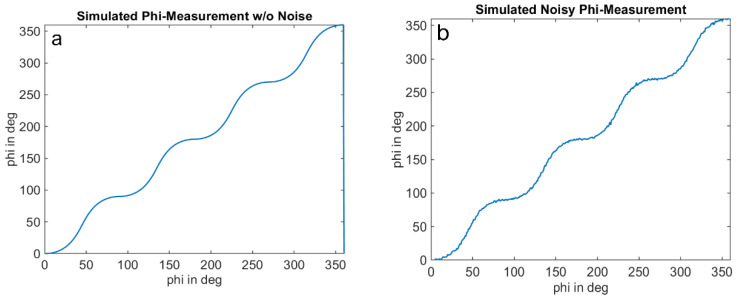
Simulation of native phase measurement of a ToF measurement using the 4-phase approach ((**a**): idealized result without simulation of noise sources, (**b**): result with added shot noise for each tap). Simulation conditions: ideal demodulation, 25% duty cycle of received optical signal, pixel full-well-capacity: 100,000 electrons, signal amplitude: 1% of the full-well, signal offset: 2% of the full-well.

**Figure 12:**
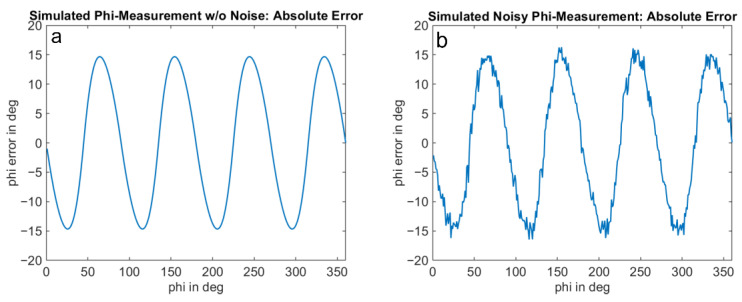
Absolute phase wiggling: Simulation of absolute error of native phase measurement of a ToF measurement using the 4-phase approach ((**a**): idealized result without simulation of noise sources, (**b**): result with added shot noise for each tap). Simulation conditions: cf. Figure 11.

**Figure 13:**
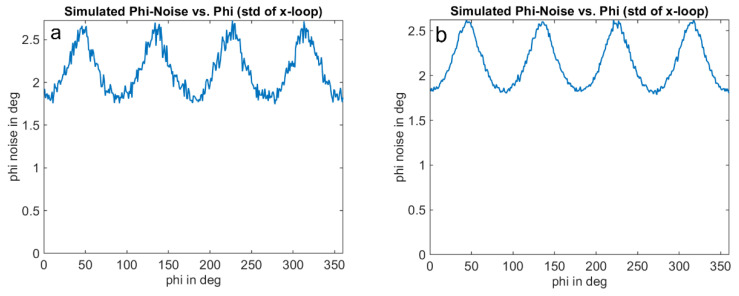
Phase noise wiggling: Simulation of expected standard deviation of ToF measurement using the 4-phase approach, with simulation conditions as of Figure 11 and Figure 12. The results represent the standard deviation of simulation of Figure 12b, repeated 500 times (**a**) and 5000 times (**b**).

**Figure 14:**
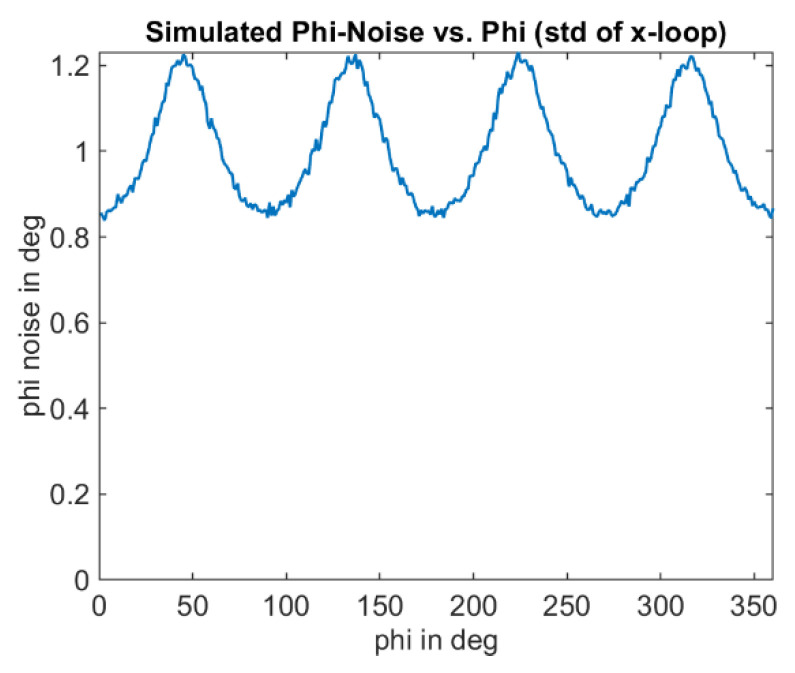
Phase noise wiggling: Simulation of standard deviation of ToF phase measurement. Same as Figure 13b but with higher signal amplitude: 2.5% of the full-well. Note that with this higher signal amplitude, the total noise level decreases, as expected.

The optical signal is emulated as a square wave signal with an adjustable duty cycle and edge steepness. In addition, the amplitude of the received signal and a signal offset can be varied as a proportion of the full-well electron count. The results for an amplitude of 1%, an offset of 2% of the full-well, and a duty cycle of the received signal of 25% are shown in Figure 11, Figure 12 and Figure 13. Figure 12 shows the well-known systematic error known as wiggling error. This error results from the ATAN evaluation of the demodulation result, which deviates from a sinusoidal function due to the actual signal shape. Figure 13, on the other hand, shows the standard deviation of the repeatedly performed measurement simulation. A systematic behavior can be seen, and a variation in the standard deviation with the measurement phase itself. We refer to this behavior as wiggling of the phase noise. Figure 14 shows the influence of the signal amplitude on the absolute value of the noise, which, as expected, decreases with higher amplitude.

In the detailed measurement series, the predicted systematic periodicity of the standard deviation with distance (phase noise wiggling) can clearly be detected. This is in good agreement with the simulation results presented in this section.

**Figure 15:**
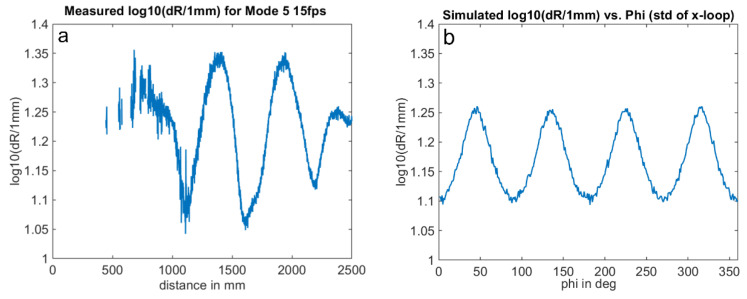
Phase noise wiggling: Variation in standard deviation dR with phase delay of received signal for constant signal amplitudes. Comparison of measurement (**a**) and simulation (**b**). Note: logarithmic scale. (simulation parameter: signal amplitude = 1% of the full-well); shortest measured distance: 0.37 m.

**Figure 16:**
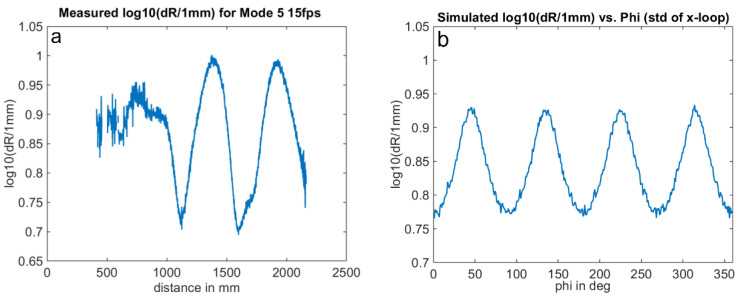
Phase noise wiggling: Variation in standard deviation dR with phase delay of received signal for constant signal amplitudes. Comparison of measurement (**a**) and simulation (**b**). Note: logarithmic scale. (simulation parameter: signal amplitude = 2.5% of the full-well); shortest measured distance: 0.37 m.

Figure 15 and Figure 16 compare the measurement results and the simulation results. The simulations were performed with signal amplitudes equivalent to the measurement. The results demonstrate a good agreement of the noise characteristics both in the absolute level of the distance noise and in their variation with the measurement phase or distance. The ToF camera used for these measurements operates in the “Mode5” setting, utilizing a modulation frequency of 60 MHz. In this mode, a distance of 2500 mm corresponds to a phase delay of 360 degrees.

## 6. Camera Model Dataset

The mathematical operations described below represent the essential components of the 3D camera model. All camera parameters used are derived from the measurements discussed earlier. The starting point is the chosen integration time $t_{\mathrm{int}}$ and the modeled 3D scene, including the camera position. In addition to their spatial location, all objects have reflectance values in the wavelength range of the 3D camera. In the first step, these objects are projected onto the image sensor using a pinhole camera calculation, resulting in a distance matrix *d*(*x*,*y*) and a reflectance matrix *r*(*x*,*y*), each with 172 × 224 elements.

Another matrix *i*_0_(*x*,*y*) with 172 × 224 elements is created, initially containing normalized intensity values per pixel. Each value corresponds to the intensity at a distance of 1078.4 mm, a reflectivity of 100% (derived from 90% measurement), and an exposure time of 510 µs, relative to the center pixel. This value has to be determined through measurements. The elements of matrix *i*_0_(*x*,*y*) are manipulated in the following steps to obtain pixel-wise intensity information that corresponds to that measured in a real 3D image acquisition of the modeled scene. In the first step, *i*_0_(*x*,*y*) is weighted with the normalized exposure time$\;t_{\mathrm{int},\;\mathrm{norm}}$.

$$t_{int,\;norm}=\frac{t_{int}}{510\;µs}\;i_{1}\left(x,y\right)=t_{\mathrm{int},\;\mathrm{norm}}\cdot i_{0}\left(x,y\right)$$

The element-wise multiplication with the distance-dependent matrix *g*(*x*,*y*) follows as the next step, where *g* can be described using the function below with the parameter b determined through measurements.

$$g\left(x,y\right)={\left(\frac{d\left(x,y\right)}{1078.4\;\mathrm{mm}}\right)}^{-b}\;i_{2}\left(x,y\right)=g\left(x,y\right)\circ i_{1}\left(x,y\right)$$

To account for illumination inhomogeneity and intensity falloff towards the edge of the sensor array, *i_2_*(*x*,*y*) is now weighted with the normalized intensity distribution *h*(*x*,*y*). We use the measured intensity distribution, normalize it to the center pixel, and factor out the variation due to increasing distance towards the sensor edge based on the flat target and the field of view.

$$i_{3}\left(x,y\right)=h\left(x,y\right)\circ i_{2}\left(x,y\right)$$

For the final intensity, *i_3_*(*x*,*y*) is multiplied by the reflectance matrix *r*(*x*,*y*).

$$i\left(x,y\right)=r\left(x,y\right)\circ i_{3}\left(x,y\right)$$

To obtain information about the error, the lookup table for noise (cf. Figure 10) is utilized. Input parameters are the intensity *i*(*x*,*y*) and distance *d*(*x*,*y*). The determined error values are stored pixel-wise in the error matrix *e*(*x*,*y*).

In an optional step, the error matrix *e*(*x*,*y*) can be used to overlay white noise onto the distance matrix, allowing the generation of a video sequence with temporal noise from these values.

**Figure 17:**
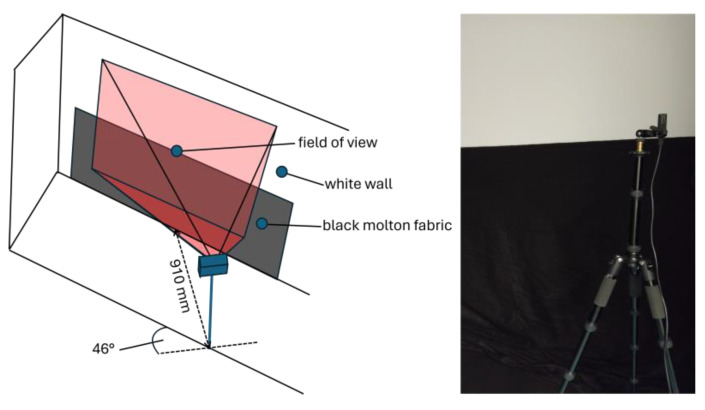
Measurement setting for evaluating the simulation data. The measurement setting consists of a flexx2 camera mounted on a tripod and positioned laterally to the surface to be measured.

To evaluate the model, we compare real-life measurement data with simulated data of the same scene. The measurement setup consists of a flexx2 camera mounted on a tripod. The camera is positioned at an angle to the surface to ensure a wide range of distances within the field of view (cf. Figure 17). The camera settings (Mode 5_15 fps; 1000 µs) were selected consistent with the measurements from Section 3. The evaluation was carried out using 100 images. The background of the field of view consists of a white wall in the upper part, and black molton fabric in the lower part. The reflection factors are approximately 90% for the white wall and 10% for the molton fabric (c.f. Figure 3).

**Figure 18:**
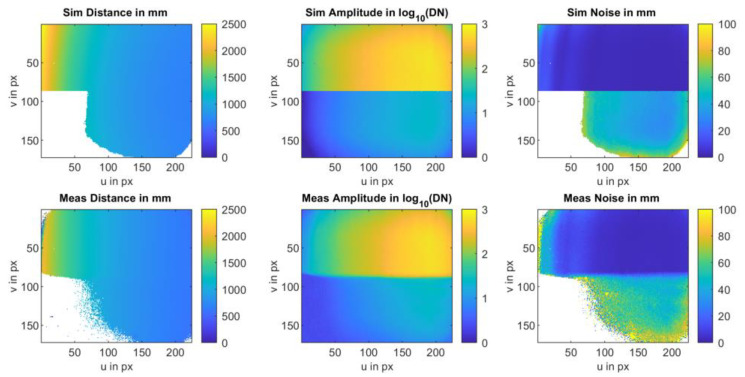
Results of the simulated (**top**) and measured (**bottom**) distance, amplitude, and noise for the experimental setting from Figure 17.

The scene, displayed in Figure 17, was then simulated using the model and compared with the results of the measurement data. The results are presented in Figure 18. The simulation determines the intensity, distance values, and the noise per pixel.

**Figure 19:**
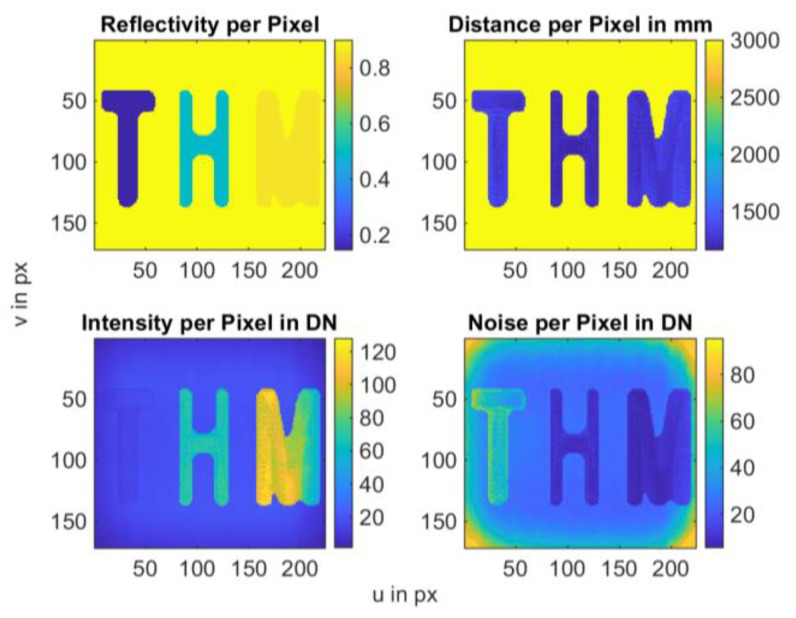
Programmatic model based on the mathematical operations and the theoretical approach (cf. Figure 1), displaying results for noise, intensity, reflectivity, and distance.

For better visualization of the mathematical model based on the theoretical model (cf. Figure 1), a programmatic model was designed using the software MATLAB, (R2023a) Version 9.14. For this model, which is presented in Figure 19, different reflection factors and distances were chosen for the letters “T, H and M” (abbreviation of Technische Hochschule Mittelhessen University of Applied Sciences). The distance between the letters and the camera is 1.2 m. Various reflection factors were selected for the letters. The grayscale value for the letter “T” is 0.15 × 256, for the letter “H” 0.5 × 256, and for the letter “M” 0.85 × 256. Pixels that are not assigned a value were subsequently provided with a fictitious background with a distance of 3 m and a grayscale value of 0.9 × 256.

## 7. Discussion

The aim of this work was the development of a theoretical model and, furthermore, the transfer of that model into a mathematical and finally programmatically executable model based on the consideration of key parameters (cf. Section 3) of AMCW-based 3D camera systems. Research interest was given to the analysis of the camera property of the wiggling, especially phase noise wiggling.

In this work, we were able to show, both by simulation and experiment, that the distance value itself, or the phase position of the AMCW measurement, has an additional systematic influence on the measurement uncertainty. Therefore, our measurements and simulations (cf. Section 5) show that the effect of phase noise wiggling can have a significant impact on noise, and thus on signal quality.

The influence of phase noise wiggling on the evaluation of 3D camera data has not been fully considered in earlier studies. In our model, it is now included in its entirety.

The programmatic model was created based on the MATLAB software program. The model provides the basis for evaluating applications for 3D recordings, considering the above-mentioned parameters. The result is a complete dataset (distance error, distance, and intensity per pixel) that can be used as a prediction model for a wide range of measurement scenarios, even before complex measurement setups must be carried out.

The camera model developed in this work can also be transferred to other camera systems, provided the following requirements are met:

(1) The camera works according to the phase-measuring AMCW method (amplitude-modulated CW), and the four phase shifts with 90° offset (four-phase method) are used.
(2) The camera parameters (number of pixels, field of view, or additional optical specifications) are known.
(3) Fulfillment of the following camera features: (a) distance and intensity information can be read out; (b) exposure time can be set; and (c) deactivation of the digital filters, and thus access to the raw data for distance and intensity, is possible.
(4) The measurement setup and the measurement system used, as well as the method of evaluation, are comparable to the one described by the authors.

The advantages of such a camera model are manifold. Application cases can be modeled in advance and the usability of ToF cameras can be evaluated. The evaluation and consideration of static parameters such as noise and amplitude are possible. Thus, specific questions, such as maximum and minimum values of distance range, reflectivities, exposure times, and frame rates, can be proactively investigated.

Particularly in a regulated measurement environment (e.g., application in a clinical setting), the model offers enormous efficiency potential through a preliminary feasibility analysis without regulatory hurdles (e.g., ethics vote).

Furthermore, 3D video data can be synthesized from the noise, distance, and amplitude data. Three-dimensional image processing algorithms can be tested and further developed based on simulated datasets. Fast simulation replaces time-consuming measurements.

If an application-specific system design of a new ToF camera is planned, the simulation model can also help to find a suitable design for the key components. The overall objective is therefore to use the camera model as a useful supplementary instrument to measurements.

Future work aims to model ambient light influence separately. This requires technical innovations, as ToF cameras typically employ narrow-band optical filters tuned to the vertical cavity surface emitting laser (VCSEL) illumination wavelength. A controlled light source with defined, homogeneous emission in the near-infrared (NIR) spectral range is necessary. While this remains a future goal, the current model is fully applicable to indoor environments with negligible NIR interference, such as those illuminated by LED.

The limitations of this work lie in the still insufficient verification of the model. We intend to investigate the verification of the model using different measurement scenarios and 3D camera systems in future publications.

The investigation of wiggling should also be examined in further research efforts with different camera systems in order to create a valid database for the evaluation of this camera property, especially since we show for the first time in this paper that wiggling not only contributes to systematic distance errors, but also leads to periodic fluctuations in the statistical measurement uncertainty that overlap with the SNR dependence.

## 8. Conclusions

This paper presents experimental measurements and simulations that show how the statistical noise behavior of modern 3D ToF cameras develops with increasing measurement distance. Based on these results, a new simulation model for 3D ToF cameras was developed that is suitable for application-specific system simulations. Previous studies assumed a linear to quadratic increase in noise with distance, depending on the noise regime. However, our results show that this model ignores a critical factor: the phase-dependent behavior of the measurement signal, which we call phase noise wiggling. The model developed in this work takes the phase noise wiggling into account for the first time. The model can be useful in a variety of applications, such as proactive investigation of specific issues of distance ranges, reflectivities, exposure times, and frame rates, or even as a partial replacement for time-consuming measurements.

## Figures and Tables

**Table 1:** Measurement modes and their utilized exposure times.

Exposure Time [%]	Mode 5_15 fps [µs]	Quantity of Measurements
100%	1020	28
90%	918	28
80%	826	28
70%	714	28
60%	612	28
50%	510	28
40%	408	28
30%	306	28
20%	204	28
10%	102	28

## Data Availability

Data are contained within the article.
